# Calcineurin and Systemic Lupus Erythematosus: The Rationale for Using Calcineurin Inhibitors in the Treatment of Lupus Nephritis

**DOI:** 10.3390/ijms22031263

**Published:** 2021-01-27

**Authors:** Carlos Rafael-Vidal, Irene Altabás, Nair Pérez, Coral Mourino Rodríguez, Jose M. Pego-Reigosa, Samuel Garcia

**Affiliations:** 1 Rheumatology & Immuno-Mediated Diseases Research Group (IRIDIS), Galicia Sur Health Research Institute (IIS Galicia Sur), SERGAS-UVIGO, 36201 Vigo, Spain; carlos.rafael@iisgaliciasur.es (C.R.-V.); irene.altabas@gmail.com (I.A.); nair.pg.89@gmail.com (N.P.); coral.mourino@iisgaliciasur.es (C.M.R.); jose.maria.pego.reigosa@sergas.es (J.M.P.-R.); 2 Rheumatology Department, University Hospital Complex of Vigo, 36201 Vigo, Spain

**Keywords:** lupus nephritis, calcineurin, T cells, clinical trial, therapeutic target

## Abstract

Systemic lupus erythematosus (SLE) is a chronic autoimmune disease with a broad spectrum of clinical presentations that can affect almost all organ systems. Lupus nephritis (LN) is a severe complication that affects approximately half of the systemic erythematosus lupus (SLE) patients, which significantly increases the morbidity and the mortality risk. LN is characterized by the accumulation of immune complexes, ultimately leading to renal failure. Aberrant activation of T cells plays a critical role in the pathogenesis of both SLE and LN and is involved in the production of inflammatory cytokines, the recruitment of inflammatory cells to the affected tissues and the co-stimulation of B cells. Calcineurin is a serine-threonine phosphatase that, as a consequence of the T cell hyperactivation, induces the production of inflammatory mediators. Moreover, calcineurin is also involved in the alterations of the podocyte phenotype, which contribute to proteinuria and kidney damage observed in LN patients. Therefore, calcineurin inhibitors have been postulated as a potential treatment strategy in LN, since they reduce T cell activation and promote podocyte cytoskeleton stabilization, both being key aspects in the development of LN. Here, we review the role of calcineurin in SLE and the latest findings about calcineurin inhibitors and their mechanisms of action in the treatment of LN.

## 1. Introduction

Systemic lupus erythematosus (SLE) is a chronic autoimmune disease with a broad spectrum of clinical presentations that can affect almost all organ systems. Kidney involvement is a common manifestation in SLE and one of the most serious complications [1]. The etiopathogenesis of SLE is complex and the mechanisms of action involved in this disease are not yet fully understood. SLE is characterized by an excessive production of nuclear debris due to aberrant and massive apoptotic events, which are detected as foreign bodies, leading to an abnormal antigen presentation that ultimately induces loss of B and T tolerance [1]. This loss of tolerance leads to T cell hyperactivation, triggering production of inflammatory cytokines, hyperactivation of B cells, massive production of autoantibodies and the formation of immune complexes, which are key in the development of lupus nephritis [2,3,4].

Calcineurin is a phosphatase involved in the production of inflammatory mediators by T cells and in the maintenance and proper functioning of the glomerular filtration membrane, which is of great importance in lupus nephritis [5,6,7]. Therefore, blocking T cells by calcineurin inhibitors (CNIs) represents a promising strategy and research and clinical trials based on CNIs have increased significantly in recent years [6,8]. This review focuses on the role of calcineurin in SLE pathogenesis and the use and mechanisms of action of CNIs as a therapeutic approach in LN.

## 2. Etiopathogenesis of Systemic Lupus Erythematosus

SLE is a worldwide pathology in which the incidence and the clinical manifestations and severity depend on different factors such as geographic area, race, gender and age. The prevalence ranges between 6.5 and 178 cases per 100,000 inhabitants and mainly affects women of reproductive age. In fact, the incidence in women is ~9 times higher than men, and the onset occurs approximately from 15 to 45 years [9,10]. Importantly for this review, kidney involvement in SLE also differs between races, with Hispanics, Mestizos, African descendants and Asians having the highest prevalence [10].

Multiple studies have shown that genetic, epigenetic and environmental processes play a key role in the onset and development of SLE. In recent years, genome wide association studies (GWAS) have associated certain genetic variants with susceptibility to the development of SLE and lupus nephritis [2,11]. Of special interest are variants in the Major Histocompatibility Genes (MHC), which are associated with the susceptibility of both SLE (*HLA-DRB1* [12]) and LN (*HLA-DQb1).* Other genes involved in lymphocyte activation and inflammation have also been associated with SLE susceptibility, such as *IKZF2* [13], *STAT4* [14], *IL-10* [15] and *TNIP1* [2,15]. Importantly, several susceptibility genes have been found by GWAS assays that are involved in the calcineurin signaling pathway, a key enzyme in the development of SLE and on which this review is focused. Specifically, an association of variants of the *NFATC1* gene, more specifically single nucleotide polymorphisms (SNP), has been found with the onset and development of lupus nephritis (LN) among SLE patients. Additionally, this gene has been associated with the development of chronic kidney disease (CKD) in the general population [16,17]. Regarding epigenetic modifications, one of the most widely described alterations is methylation. It has been found that T cells from patients with SLE present increased levels of global hypomethylation respect to healthy individuals, which is relevant from the point of view of the mechanisms of action of the disease. This allows the aberrant expression of genes that contribute to the hyperactivation and loss of tolerance of T cells seen in SLE. Likewise, it has been found that there are other epigenetic regulations that modulate the onset and development of SLE, such as the post-transcriptional regulation of certain microRNAs, as well as the histones modification [2,18,19].

Finally, the existence of an association between some environmental factors with the development of SLE has also been shown. These factors include exposure to ultraviolet light, smoking or postmenopausal hormone therapy, among others, which increase the risk of developing SLE [20]. Altogether, the involvement of environmental, genetic and epigenetic factors, as well as alterations and dysregulations of the immune system, allow SLE to be defined as a complex disease.

## 3. Characteristics of Lupus Nephritis

Approximately half of all SLE patients develop lupus nephritis (LN), which is a major cause of morbidity and mortality with high healthcare costs [21,22,23,24,25,26]. The Systemic Lupus International Collaborating Clinics (SLICC) Group reported the association in its inception cohort of LN with almost three times more risk of death and approximately 44 times more risk for the development of end stage renal disease (ESDR) [27]. Lupus nephritis is a severe complication of SLE in which the accumulation of immune complexes plays a key role [28]. LN can occur in several clinical presentations, from asymptomatic forms to rapidly progressive kidney disease, which correspond to different histological classes of lupus nephritis (LN) [29,30,31]. In addition, patients with LN are prone to present hypertension, renal failure and infections in earlier stages of the disease, among other complications [26,30]. Regarding the development of the disease, it has periods of flares alternating with periods of latency [23]. Therefore, prompt recognition and treatment of LN are mandatory in order to minimize its impact on a SLE patient. On the other hand, although there is increasing knowledge about the mechanisms involved in the development of lupus nephritis, to date it has not been possible to develop effective biomarkers, which would contribute to an earlier diagnosis or a better treatment for these patients. This is especially relevant given that, to date, there is no laboratory test that allows to replace renal biopsy, which is an invasive technique with potential complications, and is still necessary both for diagnosis and for evaluation of disease progression [32]. Furthermore, there is currently a lack of effective treatments for SLE in general and especially for lupus nephritis [33].

## 4. T Cell Dysregulation in Systemic Lupus Erythematosus

In SLE, T cells undergo an aberrant hyperactivation that induces the excessive production of several cytokines such as Interleukin (IL)-17, tumor necrosis factor (TNF), interferon (IFN)-γ and IL-21, which are involved in the induction of tissue damage, the recruitment of inflammatory cells to affected tissues and the co-stimulation of B cells. This co-stimulation triggers the hyperactivation of B cells, their differentiation into plasma cells and, lastly, a massive production of autoantibodies [2,3,4].

IL-17 is a cytokine mainly produced by T helper 17 (Th17) cells, a subpopulation of CD4^+^ T cells involved in the defense against fungi and bacteria and which has also been associated with the development of numerous autoimmune diseases [34,35,36]. IL-17 levels and IL-17-producing cells are elevated in circulation of patients with SLE [37], but also in the kidneys of patients with lupus nephritis [38]. Importantly, several studies have demonstrated that IL-17 is involved in different processes of SLE pathogenesis [37,39,40,41,42]. IL-17 promotes the recruitment of inflammatory cells to tissues, especially neutrophils. This cell type is key since neutrophils in SLE experience aberrant levels of apoptosis, a process called NETosis, which contributes to the perpetuation of cellular debris production and the activation of T and B cells [43]. Increased levels of IL-17 in SLE also induces the production of IL-6 by macrophages and monocytes and B cells proliferation and differentiation [44]. High circulating levels of TNF have been also found in patients with SLE [45,46], and its activity in this pathology is associated with its function as a growth factor for B cells and with the induction of apoptosis, as well as promoting the maturation of dendritic cells, among other functions [47]. Importantly, it has been shown that TNF could act as a biomarker of disease activity in SLE [48]. In the same way, IFN-γ and IL-21 are also elevated in circulation in SLE patients [46,49]. Moreover, expression of IL-21 has been found increased in the skin of SLE patients [50]. These cytokines contribute to the pathology, producing tissue damage and favoring the activation and differentiation of B cells, which promotes the production of autoantibodies and enhances the development of the disease [51,52].

## 5. Mechanisms Involved in T Cell Dysregulation: TCR Rewiring

The antigen presentation to T0 cells is mediated by the binding of the T cell receptors (TCR) to the major histocompatibility complex II (MHC-II) expressed by the antigen-presenting cells (APC). The proper functioning of the antigen presentation guarantees the adequate recognition by the immune system of foreign and own agents. Under normal conditions, after the synapse between the MHC and the TCR, the binding of the T cell co-receptor CD28 to CD80/CD86 on APC occurs, which allows the recognition of antigenic peptides and the subsequent intracellular signaling [53].

The TCR is a protein complex consisting of an extracellular heterodimeric region of αβ or γδ polypeptide chains associated with a CD3 region, which presents two ε, two ζ, one γ and one δ polypeptide chains [54,55]. The binding of the MHC II peptide to the binding region of the TCR induces the phosphorylation of the immunoreceptor-based tyrosine activation motif (ITAM), which is mediated by Lck, an enzyme belonging to the Src family of protein tyrosine kinases [53,56]. The phosphorylation of ITAM leads to the binding of the ζ-associated protein (ZAP-70), which initiates a cascade of phosphorylations that allows the proper signaling of the TCR [53,54,55].

In SLE patients, this TCR signaling is substituted by an alternative TCR signaling, named TCR rewiring. SLE patients present a decrease in the CD3-ζ chain levels, through transcriptional, post-transcriptional and post-translational regulatory mechanisms [57,58,59,60,61]. Remarkably, T cells from SLE patients transfected with TCR ζ chain reversed the TCR signaling abnormalities [62]. Hypothetically, the reduction in ζ chain levels would trigger the inactivation of this signaling. However, several works have shown an opposite role [3,60]. This is due to the fact that the ζ chain is replaced by Fc receptor gamma (FcRγ), which binds to the spleen tyrosine kinase (Syk). Syk triggers a signaling of greater efficiency than ZAP-70, leading this manner to the hyperactivation of T cells in SLE [3,58,60,63]. Importantly, the elevated Syk expression observed in SLE patients [63] also contributes to the higher activation of T cells. Syk kinase activation induces the phosphorylation of different protein tyrosine kinases, with one of the most important being the phospholipase Cγ (PLCγ). The activation of PLCγ leads to the hydrolysis of phosphatidylinositol-4,5-bisphosphate (PtdIns(4,5)P2) in the membrane, inducing in this manner the release of inositol-1,4,5-trisphosphate (InsP3) and diacylglycerol (DAG), which play a key role in the signaling process. On the one hand, InsP3 migrates to the endoplasmic reticulum, where it binds to the InsP3R receptor and induces the release of calcium into the intracellular space. On the other hand, the release of DAG in the membrane promotes the activation of membrane channels that allow the entry of calcium from the extracellular space. Among these channels are calcium release-activated channels (CRAC), Transient Receptor Potential (TRP) channels and voltage-gated Ca^+2^ channels (VOCC). Finally, the intracellular calcium binds to calmodulin and leads to activation of calmodulin-dependent calcineurin phosphatase, shortly known as calcineurin [51,54,60,61,64,65], which plays a key role in the pathogenesis of SLE.

## 6. Calcineurin Role and Characterization

Calcineurin is a serine-threonine phosphatase heterodimer consisting of two different subunits. The subunit A, which is the largest and is responsible for binding to calmodulin, presents a catalytic site, a calmodulin-binding domain and an autoinhibitory domain. On the other hand, the B subunit contains four calcium binding regions and is defined as the regulatory subunit [66,67].

**Figure 1:**
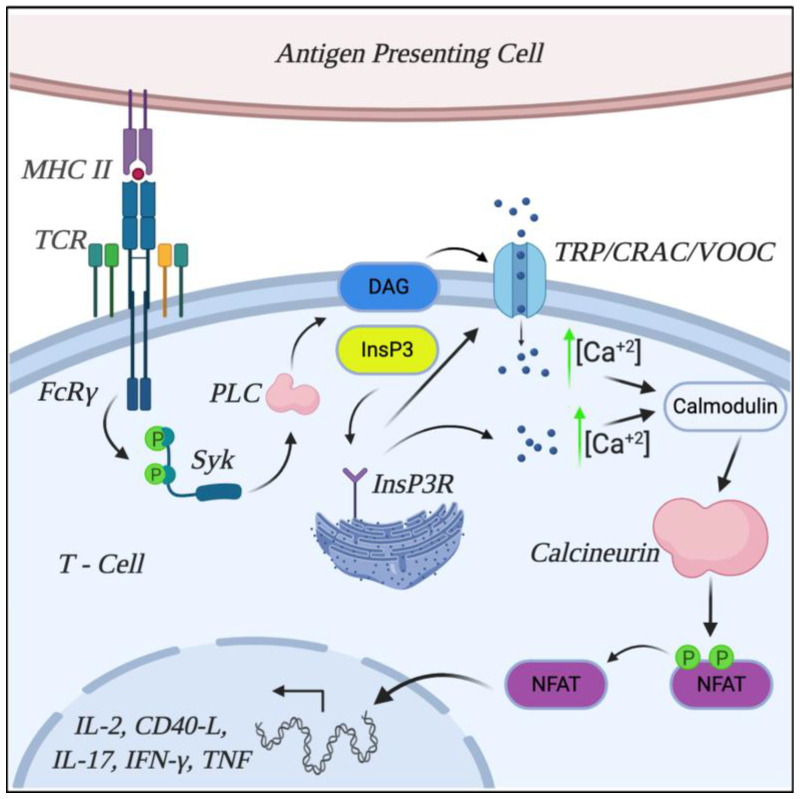
T cell receptors (TCR) signaling and the role of calcineurin in systemic lupus erythematosus. After antigen presentation to T0 cells in SLE patients, TCR rewiring occurs, so the ζ chain is replaced by Fc receptor gamma (FcRγ), which binds to the spleen tyrosine kinase (Syk) and induces the phosphorylation of the phospholipase C (PLC). This phosphorylation induces the release of inositol-1,4,5-trisphosphate (InsP3) and diacylglycerol (DAG). The release of DAG in the membrane promotes the activation of membrane channels calcium release-activated channels (CRAC), Transient Receptor Potential (TRP) channels and voltage-gated Ca^+2^ channels (VOCC), allowing the entry of calcium from the extracellular space. Then, InsP3 migrates to the endoplasmic reticulum, where it binds to the InsP3R receptor and induces an influx of calcium into the intracellular space. The intracellular calcium binds to calmodulin and leads to activation of calcineurin phosphatase. Calcineurin dephosphorylates the nuclear factor of activated T-cell (NFAT) and induces the translocation of this transcription factor to the nucleus, where it promotes the expression of several key genes such as Interleukin (IL)-2, IL-17, tumor necrosis factor (TNF), interferon (IFN)-γ and CD40-L(igand).

Following the calcium- and calmodulin-mediated activation, calcineurin dephosphorylates the family of nuclear factors of activated T-cell (NFAT), which consists of five members (NFAT1-5), of which only NFAT1, NFAT2 and NFAT4 are expressed in T cells [68]. The calcineurin-dependent NFAT dephosphorylation induces the translocation of this transcription factor to the nucleus, where NFAT regulates the expression of several key genes. Here, NFAT members bind to DNA and induce the expression of different proinflammatory cytokines and proteins, mainly TNF, IL-2, CD40 ligand (CD40-L), IL17 and IFNγ, highly implicated in the pathogenesis of SLE [2,55,69,70,71,72]. This signaling process is summarized in Figure 1. Thereby, T-cell hyperactivation mediated by aberrant TCR signaling leads to increased calcium levels, hyperactivation of calcineurin and, ultimately, increased inflammatory mediators that contribute to the development of SLE.

In addition to the role of T cell hyperactivation, calcineurin is involved in the stabilization of the glomerular filtration membrane function, specifically in the podocytes [7]. Podocytes are epithelial cells with a well-developed cytoskeleton that are essential for the proper working of the glomerular filtration barrier in the kidneys [73]. Several alterations have been observed in the podocytes of lupus nephritis patients, which contribute to proteinuria and kidney damage [74]. Importantly, calcineurin acts on different podocyte signaling pathways, which contribute to kidney damage. On the one hand, calcineurin is involved in the synaptopodin-mediated cytoskeleton destabilization. In the absence of calcineurin or when it is blocked, synaptopodin is phosphorylated and binds to the protein 14-3-3, which stabilizes the podocyte cytoskeleton. However, the activation of calcineurin dephosphorylates synaptopodin, leading to its cleavage mediated by cathepsin L and finally triggering the destabilization of the podocyte cytoskeleton. On the other hand, calcineurin dephosphorylates the Bcl-2-associated death promoter (BAD) and the dynamin-related protein 1 (Drp1), which promotes podocyte apoptosis [75,76,77,78]. Related to this, it has been reported that there are increased levels of calcium in podocytes in SLE and its entrance is mainly mediated by the TRPC-6 ion channel, which has been shown to be involved in the alteration of podocytes in numerous kidney pathologies [75]. In this regard, an increase in TRPC-6 mRNA levels in the urine of patients with active lupus nephritis has recently been reported [79].

## 7. Calcineurin Inhibitors: Characterization and Mechanisms of Action

The key role of calcineurin on the hyperactivation of T cells and the alteration of podocytes phenotype postulates these signaling pathways as a therapeutic strategy for the treatment of SLE patients. Remarkably, the molecular structure of the CNIs, as the high lipophilic character of the CNI allow them to easily reach fatty organs such as the adipose tissue, lymph nodes and, interestingly, the kidneys [80].

Currently, there are two CNIs: cyclosporine-A (CsA) and tacrolimus (TAC), which have an immunosuppressive role and are widely used in clinical practice to treat autoimmune diseases and to prevent organ rejection [80]. A third CNI, Voclosporin (VCS), has demonstrated its superiority in comparison to placebo in the treatment of LN and will probably be licensed for that indication [81].

CsA is an immunosuppressant and lipophilic molecule that is chemically defined as a cyclic endecapeptide [82]. Regarding its mechanism of action, owing to its lipophilic character, CsA enters to the cytoplasm and binds to cyclophilin, a cytoplasmic receptor belonging to immunophilins, which are a family of intracellular receptors that are able to bind to calcineurin. Thereby, CsA binds to cyclophilin and this immunosuppressant complex is capable of inhibiting the calcineurin activity. Therefore, CsA acts as a CNI in a cyclophilin dependent manner [83,84].

TAC is a lipophilic macrolytic lactone, structurally different from CsA and, therefore, its mechanism of action is also different. Importantly, the immunosuppressive potency of TAC is significantly higher than observed for CsA [83,85]. Mechanistically, intracellular TAC binds to FK binding proteins (FKBP), especially FKBP12. FKBP, similarly to cyclophilins, belong to the immunophilin family, and have a higher affinity for calcineurin in the presence of ligands. Therefore, the TAC-FKBP complex binds to calcineurin and inactivates its enzymatic activity, halting the production of cytokines and proinflammatory and costimulatory molecules [83,86].

VCS is an analog of CsA, but differs in chemical structure due to a modification in the functional group of the amino acid residue in position 1. This modification is of great relevance, since it confers an immunosuppressive effect significantly higher to that of CsA [87,88,89]. VCS binds, similar to CsA, to cyclophilin in the cytoplasm. However, it does not do it in the same way as CSA, but the modification of its chemical structure gives it a greater affinity for cyclophilin-A and, in turn, a greater effectiveness than CsA in the inactivation of calcineurin and in its immunosuppressive role [90].

Therefore, these three calcineurin inhibitors are able to block the pathways in which calcineurin is involved, such as the hyperactivation of T cells and the phenotypic alteration of podocytes. This inhibition finally leads to the reduction of pro-inflammatory mediators (IL-2, IL-17, TNF, IFN-γ and CD40-L) secreted by T cells [62,79,82] and to the cytoskeleton stabilization and inhibition of apoptosis in podocytes [75,76,77], pointing out the use of CNI for the treatment of LN. CNIs also reduce proteinuria through nonimmune mechanisms such as stabilizing the cytoskeleton and preventing podocyte apoptosis [91]. Although this can be of benefit in LN, that fact may cause uncertainty in a clinical or investigational setting if the proteinuria level is included in the outcome measure (partial or complete remission) as CNIs can mask proteinuria in presence of ongoing renal activity or damage. In addition, as SLE mainly affects women of childbearing age, one of the main clinical advantages of CNIs is the fact that their use is compatible with pregnancy and breastfeeding so ciclosporin A and tacrolimus can be used to prevent or manage LN flares during these periods [92,93,94].

The main concern of CNIs is their potential nephrotoxicity. Acute nephrotoxicity is due to vasoconstriction of the afferent renal arterioles and is reversible after CNI dosage reduction or withdrawal [95]. Chronic CNI renal toxicity has a complex pathogenesis and presents as a progressive loss in glomerular and tubular function [96]. Tacrolimus is less nephrotoxic than CSA due to its weaker vasoconstrictive effect and lower fibrogenic effect [6].

On the other hand, CNIs have a narrow therapeutic range, so it is necessary to monitor drugs levels in order to ensure their effectiveness and safety [6]. Although rare and more frequently associated with their use in renal transplantation, CNIs can induce microangiopathic hemolysis that may worsen the prognosis of the patient [97].

## 8. Calcineurin Inhibitors in Lupus Nephritis

Goals of treatment in LN should aim at remission, prevention of flares and organ damage with the fewest drug side-effects [98,99]. The European League Against Rheumatism (EULAR)/European Renal Association (ERA)-European Dialysis and Transplant Association (EDTA) recommendations for the management of LN recommends initial induction treatment in class III–IV LN with mycophenolate mofetil/mycophenolic acid (MMF/MPA) or cyclophosphamide (CYC), considering some ethnic and demographic characteristics, in combination with glucocorticoids. As maintenance immunosuppressive treatment, MMF/MPA or azathioprine (AZA) are considered as standard of care [99].

The use of CNIs has been reserved as a second line treatment in non-responders or refractory disease, particularly in patients with high level of proteinuria with proliferative LN, membranous LN or podocytopathy, due to its powerful antiproteinuric effect [98,99]. However, several studies published over the last years suggest that CNIs may be efficacious to treat not only refractory but newly diagnosed LN patients so they may an attractive option for the management of lupus nephritis [76,81].

In the Cyclofa-Lune Study, 40 patients with active proliferative LN were randomized to receive sequential induction and maintenance treatment either with CsA or CYC [100]. There were no statistical differences in remission in response between the two treatment regimens, with similar rates of ESRD at the end of the study [100]. Moroni et al. [101] have also demonstrated the effectiveness of CsA for maintenance therapy in LN [101]. Argolini et al. [102], in a multicentric retrospective study compared CsA, MMF and AZA in maintenance therapy in 106 LN patients after eight years of follow up. They did not find any differences in achieving or maintaining complete renal response, although patients in the CsA group had more severe baseline clinical features, such as higher proteinuria levels. There were no differences in the severity of side effects between groups [102].

Most of the data about TAC in LN treatment come from studies in the Asian population [103,104,105]. In a non-randomized prospective cohort study, 40 patients with diffuse proliferative or membranous LN were treated either with TAC for induction and maintenance for 12 months or with CYC for 6 months and maintenance with AZA for other 6 months [103]. The main findings of the study were a significant reduction in proteinuria in the first month and a higher incidence of complete renal remission (CRR) by 5 and 12 months in the TAC group compared to the CYC group, with less severe side effects. Mok et al. [105] carried out a larger trial with 150 LN patients randomized to receive TAC or MMF for induction, concluding that TAC was not inferior to MMF, although there was a non-significant tendency to more incidence of renal flares and renal function decline in the TAC group.

The multitarget therapy, a combination of MMF and TAC, is an alternative option for induction therapy in LN patients with high range proteinuria [99]. This is supported by the results of various clinical trials, which show higher CRR rates in LN patients treated with multitarget therapy compared to CYC without an increase in adverse events and similar rates of relapse [106,107,108].

Three meta-analysis reviewed the use of CNIs alone or in multitarget therapy, compared to intravenous MMF and CYC in LN [109,110,111]. The first one, which included nine studies and was published in 2016, evidenced that TAC alone as induction therapy is associated with higher CRR rates compared to intravenous CYC (*p* = 0.004), while there were no significant differences compared to MMF [109]. On the other hand, the multitarget therapy was more effective than intravenous CYC only when partial remission was included (*p* = 0.0006) [109].

The second meta-analysis was also published in 2016 and included 23 studies, of which six were randomized controlled trials (RCTs), all of them conducted in patients of Asian ethnicity [110]. These RCTs analyzed TAC as induction therapy (five times) and as maintenance therapy (once), showing that TAC regimens achieved a significantly higher total response and significantly higher complete response (*p* < 0.05) than those with CYC or MMF without TAC [110].

The third meta-analysis included 53 studies and compared the efficacy and toxicity of the different first-line treatments for LN [111]. It concluded that the multitarget therapies, CNIs and MMF, were more effective than intravenous CYC to induce remission of LN and that MMF was the most effective treatment to maintain remission [111].

Of special interest is the use of TAC in LN renal transplant patients, due to the fact that, despite the recommended therapeutical regimes, LN can lead to end stage renal disease and renal transplantation. An incidence of renal transplantation due to LN of 3 to 4 per million per year has been described in the US [112]. An estimated incidence of LN recurrence in kidney allografts of 2% to 11% has been reported after a median follow-up duration of 4 years [113]. TAC is also used to prevent rejection in renal transplant patients [114], but there is no evidence that this fact can have any influence on the chance of LN recurrence.

VCS is a novel CNI under study for the treatment of LN. AURA-LV is a Phase 2, randomized, double-blind, placebo-controlled trial that compared two doses of VCS plus MMF versus placebo plus MMF for induction of remission in LN [115]. The primary endpoint was CRR at 24 weeks. This was achieved in a significatively higher percentage of patients in the VCS group compared to the placebo group. The significantly greater CRR rate was maintained at 48 weeks. More severe adverse events were seen in both VCS groups [115]. AURORA is a Phase 3, randomized, double-blind, placebo-controlled trial that compared VCS to placebo in addition to MMF and rapidly tapered oral glucocorticoids in 357 patients with active LN [81]. The primary endpoint was achieving CRR, defined as urinary protein/creatinine ratio of ≤0.5 mg/mg, with an estimated glomerular filtration rate (eGFR) ≥60 mL/min/1.73 m^2^ (or no confirmed decrease from baseline in eGFR of >20%, presence of sustained, low dose steroids and no administration of rescue medication) at 52 weeks. The CRR rate was significantly greater at week 52 in the VCS group compared to the placebo group (40.8% vs. 22.5%, respectively; *p* < 0.001). Ethnicity subgroup analysis demonstrated that the greater efficacy of VCS compared to placebo was also maintained (38.6% vs. 18.6%, respectively; *p* = 0.0062) in Hispanic/Latino patients, a difficult to treat group.

## 9. Concluding Remarks

In this review, we have characterized the biochemical aspects and the mechanism of action of CNIs. These inhibitors are able to block the hyperactivation of T cells and allow the stabilization of podocytes, both critical processes in the development of LN, thereby improving the condition of these patients. On the other hand, our review highlights the growing evidence that well-designed clinical trials support the role of CNIs in the treatment of LN. In addition, it could also be a potential approach for other autoimmune diseases characterized by the excessive activation of T cells such as SLE patients without renal involvement, rheumatoid arthritis, multiple sclerosis, psoriasis, psoriatic arthritis, Behcet’s disease and atopic dermatitis, among others [116,117,118,119,120,121]. In fact, trials have already been carried out in some of these diseases (clinicaltrials.gov identifiers: NCT00209859; NCT00377325; NCT00167583 [122,123,124]). Likewise, the stabilizing activity of the CNIs in the podocytes postulates this therapeutic strategy as a potential treatment for other kidney diseases in which the podocytes are affected, which may lead to chronic renal failure. In conclusion, CNIs represent an innovative and successful strategy for the treatment of LN, a condition for which there are unmet needs in terms of therapies. CNIs are also a potential treatment for other diseases in which T cells are involved. All this shows that CNIs provide useful treatment for a wide spectrum of systemic autoimmune diseases.
